# Determining areas that require indoor insecticide spraying using Multi Criteria Evaluation, a decision-support tool for malaria vector control programmes in the Central Highlands of Madagascar

**DOI:** 10.1186/1476-072X-6-2

**Published:** 2007-01-29

**Authors:** Fanjasoa Rakotomanana, Rindra V Randremanana, Léon P Rabarijaona, Jean Bernard Duchemin, Jocelyn Ratovonjato, Frédéric Ariey, Jean Paul Rudant, Isabelle Jeanne

**Affiliations:** 1 Cellule Système d'Information Géographique, Unité Epidémiologie, BP1274, Tel 261 20 22 412 72 Institut Pasteur, Antananarivo, Madagascar; 2 CERMES, BP 10887 Niamey, Niger; 3 Institut Pasteur, Phnom Penh, Cambodia; 4 Institut Francilien de Géosciences, Université Marne La Vallée, France

## Abstract

**Background:**

The highlands of Madagascar present an unstable transmission pattern of malaria. The population has no immunity, and the central highlands have been the sites of epidemics with particularly high fatality. The most recent epidemic occurred in the 1980s, and caused about 30,000 deaths. The fight against malaria epidemics in the highlands has been based on indoor insecticide spraying to control malaria vectors. Any preventive programme involving generalised cover in the highlands will require very substantial logistical support. We used multicriteria evaluation, by the method of weighted linear combination, as basis for improved targeting of actions by determining priority zones for intervention.

**Results:**

Image analysis and field validation showed the accuracy of mapping rice fields to be between 82.3% and 100%, and the Kappa coefficient was 0.86 to 0.99.

A significant positive correlation was observed between the abundance of the vector *Anopheles funestus *and temperature; the correlation coefficient was 0.599 (p < 0.001). A significant negative correlation was observed between vector abundance and human population density: the correlation coefficient was -0.551 (p < 0.003). Factor weights were determined by pair-wise comparison and the consistency ratio was 0.04. Risk maps of the six study zones were obtained according to a gradient of risk. Nine of thirteen results of alert confirmed by the Epidemiological Surveillance Post were in concordance with the risk map.

**Conclusion:**

This study is particularly valuable for the management of vector control programmes, and particularly the reduction of the vector population with a view to preventing disease. The risk map obtained can be used to identify priority zones for the management of resources, and also help avoid systematic and generalised spraying throughout the highlands: such spraying is particularly difficult and expensive.

The accuracy of the mapping, both as concerns time and space, is dependent on the availability of data. Continuous monitoring of malaria transmission factors must be undertaken to detect any changes. A regular case notification allows risk map to be verified. These actions should therefore be implemented so that risk maps can be satisfactorily assessed.

## Background

The highlands of Madagascar present an unstable pattern of transmission of malaria. The population has no immunity and there have been epidemics with large numbers of casualties [1,2]. The last epidemic occurred in the 1980s and killed about 30,000 people [3]. The epidemics in Malagasy highlands corresponded to the re-emergence of malaria and spread after the vector control programme was reduced or stopped [4]. The same phenomenon has been observed in Belize, Central America and highlands in Africa [1,4,5]. The malaria epidemic control strategy in the Malagasy highlands has been based on indoor insecticide spraying, to control the vectors. *Anopheles funestus *(*An. funestus*) is the main vector responsible for malaria epidemics in the Central Highlands of Madagascar [1]. The success of the vector control programme was due to the anthropophilic and endophilic behaviour of *An. funestus*.

During the first cycle of the vector control programme, the major criterion used for the inclusion of a village was simply being at an altitude between 1,000 m and 1,500 m [6]. This resulted in huge areas needing to be covered by indoor insecticide spraying during each of the five cycles of the programme named "Opération de Pulvérisation IntraDomiciliaire" (OPID). This was followed by annual selected indoor insecticide spraying named "Campagne d'Aspersion IntraDomiciliaire" (CAID) based on alerts declared by Health Facility Centres and confirmed biologically by epidemiological surveillance centres, named "Poste Sentinelle de Surveillance Epidémiologique (PSSE)". Complete coverage of the highlands by indoor insecticide spraying programmes would require a massive logistical effort: a single campaign may cost as much as US $3,500,000. Furthermore, the human resources are not available, some of the areas are inaccessible and to be effective, the programme must be implemented in a relatively short time window, immediately before the malaria transmission season. Thus, it would be extremely beneficial to select priority zones allowing action to be better focused. Generalised spraying could be avoided, and more selective, targeted treatment used.

In Kenyan highlands, malaria is associated with ecological risk factors. Higher elevation, increased distance from the forest and swamp and increased population density were associated with decreased malaria risk [7].

The objective of the study is to determine zones at risk of malaria epidemic using Geographic Information System and then to guide the vector control programmes. Finally, the results of this study could be used as a decision – support tool for the decision makers in resource management.

## Methods

### Study zones

**Figure 1:**
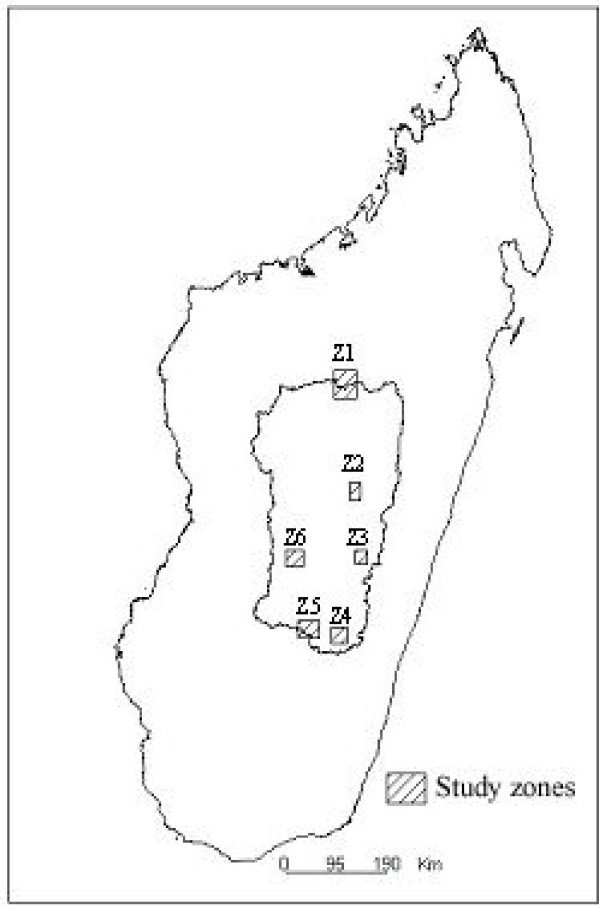
Geographical location of the study zones in the central highlands of Madagascar.

Six zones, numbered z1 to z6 (hatched surfaces in figure 1), were chosen according to their geographical location in the central highlands, the dominance of rice field typology, the parasite prevalence among schoolchildren as determined in 1998 [6] and the time of the last indoor insecticide spraying campaign.

Mahatsinjo (zone 1): This zone is located on the Northern margin of the Highlands. The rice fields occupy the small valley bottoms. This area had never been treated with insecticide spraying.

Ambohibary (zone 2): This is a vast plain of rice fields on the North Eastern part of the mountain of Ankaratra, at more than 1600 m above the sea level. This area has been treated with only the first cycle of insecticide spraying.

Ambositra (zone 3): This zone is in the Andina valley. Rice fields occupy broad valleys and the micro-basins managed in regular parcels. The lower closed slopes are managed for dry cultivation or in terraces around the hills for rice fields. This area has been treated with insecticide spraying.

Ambalavao (zone 4): This zone is at the southern edge of the central highland. Long digitated rice fields cover the secondary valleys and the small tertiary valleys. This zone has been treated with insecticide spraying.

Fenoarivo (zone 5): In the district of Ambalavao, this zone includes the same types of rice fields found in zone 4. In particular, rice fields occupy the small valleys and also pluvial micro-parcels. Most of this zone is at less than 1,000 m altitude and has never been treated with insecticide spraying.

Amborompotsy (zone 6): This zone is situated in the west part of the central highlands. Smooth slopes to deep valleys are managed in terraces for rice cultivation but wider valleys are only exploited at the bottom. Amborompotsy has never been treated with insecticide spraying.

### Data sources

- A Digital Elevation Model (DEM) was obtained for all the highlands of Madagascar (between 45°26E and 48°05E of longitude, 17°39S and 22°12S of latitude), from the Earth Observation Epidemioproject of the European Spatial Agency (ESA). The horizontal ground resolution was 50 m and the map scale was 1: 200 000.

- Temperature distribution was determined with the Spatial Characterization Tool (SCT) [8].

- Population density was obtained from the Ilo-Cornell University, FoFiFa (Foibe Fiofanana momba ny Fambolena) and Instat (Institut National des Statistiques) project in 2001. This was used to update the data from the last population census that was in 1993.

- Enhanced Thematic Mapper+ of Landsat 7, preprocessing level 1G (resolution 30 m) was provided by USAID through the National Geographical and Hydrographical Institute of Madagascar.

- XI images of Spot 4 (pre-processing level 2A, resolution 10 m) were provided by the Centre National d'Etude Spatial (CNES) and the SIGREP project (Système d'Information Géographique pour la prévention du Risque de survenue d'Epidémie de Paludisme), on the highlands of Madagascar. The SIGREP was conducted by malaria research group of Institut Pasteur de Madagascar.

- Synthetic Aperture Radar Precision Images (SAR PRI) from ERS2 and Advanced Synthetic Aperture Radar Image Mode Precision images (ASAR_IMP_1P) from Envisat, were acquired from the Project CAT1 – 2320 of the European Spatial Agency (ESA) through the Action Concertée Inter Pasteurienne (ACIP) project. Synthetic Aperture Radar (SAR) and Advanced Synthetic Aperture Radar (ASAR) images were used for Amborompotsy (z6). The image from ERS2 was acquired in May 2003 and that from Envisat was acquired in January 2004. Two ASAR images were used for Fenoarivo (z5): the first image was acquired in January 2004 and the second in July 2004.

- Delimitations of the "communes" (administrative districts) were acquired from the National Geographical and Hydrographical Institute of Madagascar.

- Insecticide spraying and malaria epidemic alert data were obtained from the Malaria control service of the Malagasy Ministry of Health and Family Planning.

### Determination of rice fields using remotely sensed data

The principal breeding sites of *An. funestus *are rice fields [3,9,10]. Topographic maps of rice fields available before our study were out-dated. Consequently, remote sensing using high spatial resolution images was used to identify larval habitats [11].

Rice field maps were updated from recent images of Landsat 7 and Spot 4. Radar images of Envisat were also used. The first fieldwork and visual interpretation of images were used to determine, for each land cover class, areas for training sites and those for tests. Rice field parcel sizes were small, so small training sites were used to avoid digitising mixed parcels. Three classes of land cover were considered: rice fields, vegetation and others (including urban areas, bare soil and water bodies). Delays in the rice cultivation schedule were observed in Ambohibary (z2) and Ambositra (z3) so two rice field classes were taken into account for training sites determination. Supervised classification by the method of maximum likelihood was used to map rice fields from optical data of six study zones. Secondary fieldwork was used to validate the maps on the ground. The post-classification process involved grouping different stages of rice fields into a "rice fields" class and all the rest (vegetation and others) into the "not rice fields" class. Isolated pixels were removed after classification. The classification was evaluated with the confusion matrix and Kappa index.

Radar image enhancement involved reducing speckle while preserving the image accuracy. Adaptive filters including a gamma and local sigma filter were applied with different sizes to optimise quality for visual interpretation. The analysis was based on the comparison of backscatter coefficients on two different dates. Regions of interest were digitised and pixel value was studied statistically to define the threshold corresponding to the class. Rice field maps obtained with optical image classification were improved by radar images for zones 5 and 6 (Fenoarivo and Amborompotsy).

### Entomological study

Entomological study was conducted in 27 villages including villages in each of the six study zones at the end of rainy season (from March to May): 1999 for Ambalavao (z4), 2000 and 2001 for Fenoarivo (z5), and 2001 for Mahatsinjo (z1), Ambohibary (z2), Ambositra (z3) and Amborompotsy (z6).

Human landing collections and residual resting mosquito fauna collections were used as standardized sampling methods. Mosquitoes that landed on the collectors' exposed legs were collected with a glass tube and a flashlight. The residual resting mosquito fauna (mosquitoes at rest) was sampled early in the morning in eight houses per village for two or three days, by pyrethrinoïd spraying. Mosquito aggressiveness was estimated by number of bites/human/night. The abundance of *An. funestus *was estimated by number of mosquitoes/house/night. Circum Sporozoite Protein ELISA tests were used to determine the infected mosquito rate. The Entomological Inoculation Rate (number of infective mosquito bites) was considered as an overall indicator of the level of malaria transmission, although determining the transmission level was not the aim of the study.

### Multi-Criteria Evaluation

The method of Weighted Linear Combination (WLC) was used to assess the weightings for factors, and to map the risks in the various zones included in the study. The MCE process included four steps: i) establishing the criteria (factors and constraints), ii) standardizing the factors, iii) establishing their relative weights, and iv) conducting the final MCE [12].

The first step of MCE is the factors determination, such as: temperature, human population density, time since last indoor insecticide spraying, distance from rice fields and surface area of rice fields per "commune". The last two factors were determined from the results of rice field determination using remotely sensed data. Proximity of potential breeding sites was calculated considering 1,000 m from rice fields as the probable flight range of female mosquitoes. The non-parametric Spearman test was used to study correlation between the abundance of *An. funestus *and the factors previously cited.

The factors present different units. They were standardized to a continuous common numeric range on a 0 to 255-byte scale. Fuzzy method was used for their standardization. Fuzzy set membership is the result of data classification according to the possibility of belonging to a class in which the boundaries between classes are not distinct. Consequently, the transition between membership and non-membership of the area is gradual.

The technique developed by Saaty (1977) was used for factor weighting [13]. This is a decision-making procedure known as the Analytical Hierarchy Process (AHP). It includes a pair-wise comparison in which only two variables were considered at a time. The continuous rating scale of Saaty (1977) was used to rate factors with the following values, 9 (extremely important), 7 (very highly important), 5 (highly important), 3 (moderately important) and 1 (equally important).

Inversely, less important variables were rated 1/3 (moderately less important), 1/5 (much less important), 1/7 (very much less important) and 1/9 (extremely less important). The pair-wise comparison table was completed with the values corresponding to the degree of importance of the factors based on the correlation study. For example, if the temperature was extremely more important than indoor insecticide spraying, one could enter 9 on the scale; and consequently indoor insecticide spraying would be extremely less important than temperature, so 1/9 would be entered.

Factors weights were calculated from the table of pair-wise comparisons and scaled with Idrisi Version 3.2 release 2 (Clark University, 950 Main street Worcester USA). The weights have to sum to one as required by the WLC procedure. The ratio consistency was calculated as the ratio of the index of coherence in the initial matrix to the random index of the matrix with the same dimension. It indicated the probability that the matrix ratings were randomly generated. If the ratio consistency is higher than 0.10, pair-wise comparison had to be re-evaluated.

Two classes were considered for altitude: the first class is suitable for epidemic (altitude between 1,000 m and 1,500 m); the second class is unsuitable for epidemic. For the second class, the transmission is continuous (altitude lower than 1,000 m) or absent (altitude higher than 1,500 m). Altitude was considered as a constraint. Constraints were defined as variables describing the properties of entities that limit their selection.

After establishing the factors and constraints, the GIS analyst generally worked in a group with the decision makers to fill out the pair-wise comparison matrix for the WLC. The assigned rating had to be justified by group members. Discussions can lead to suggestions for different ratings [12]. Here, the weighting of malaria transmission factors was based on the formal results of the correlation study between vector abundance and malaria transmission factors.

The WLC procedure was to multiply each factor by its weight, adding the results and then successively multiplying the result by each of the constraints [12]. The risk maps have a range of 0 – 255.

S_i _= Σ((W_1_F_1i_,...., W_k_F_ki_)/Σ(W_1_, ..., W_k_)) ∩ C_1-c,i_

Where:

S_i _= Final score for cell i

F_1-k _= Cell property for the factor 1 to k

W_1-k _= Factor weight 1 to k

C_1-c _= Cell property for the constraint 1 to c

### Validation

At the end of the OPID cycle, an epidemiological surveillance and malaria alert system on the Highlands was implemented in 1998. This involved PSSE with microscope facilities for malaria diagnosis, each under the responsibility of a physician. The microscopic diagnosis is rarely available in the health facility centres. The surveillance is based essentially on routinely notified cases of suspected malaria (temperature >37.5°C, no clinical signs of other disease). A monthly alert threshold was proposed for all the health facility centres. This threshold is calculated as the monthly average of presumed malaria cases during the five years protected by OPID, plus two standard deviations.

If the threshold is exceeded, the district health service informs the PSSE doctor, who then conducts an epidemiological investigation. The main steps of this investigation are: (i) confirming that the criteria for the definition of cases of suspected malaria cases have been respected; (ii) microscopic confirmation of the presence of the parasite [14].

The risk maps were validated with the results of the investigation in health facility centres, which had posted an alert. Only 13 such centres were included in the study zones, and were defined as the villages in which they operate. These villages were superimposed on the risk map.

For zones with an altitude lower than 1,000 m, transmission is continuous; a confirmed alert indicates a good agreement with the risk mapping. Similarly, for zones with an altitude higher than 1500 m, transmission can be considered to be zero, so an alert that is subsequently not confirmed agrees well with the risk map. The zones between 1,000 m and 1,500 m are considered to be those at risk of epidemics. The maps obtained show a risk gradient. Non-confirmed alerts in zones scored as low risk, and confirmed alerts in zones scored as high risk, were both considered indicating agreement.

## Results

### Determination of rice fields

Topography had an effect on the quality of SAR and ASAR images so automatic classification was very difficult. Vegetation, rice fields and some moors gave the same radiometric response in the images of Spot 4. However, radar images separated moor and vegetation. These features (flooded and mixed parcels) made correct determination of rice fields difficult. Most of the land surface showed varied cultivation. The parcels detected were too small and the rice fields were also intermingled with reed beds. Distortion due to the remoteness of the sensor data compounded these problems. It impaired image enhancement and automatic classification. To compromise between reduction of speckle and conservation of accuracy, various image filters were compared especially for detected objects that were very small.

**Figure 2:**
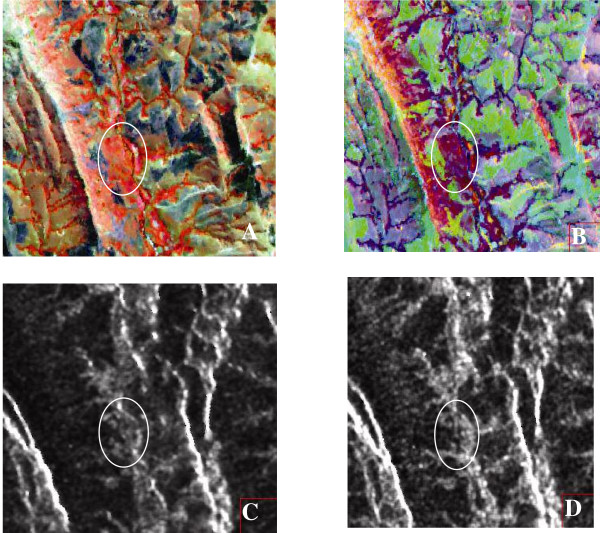
2A Image of Spot 4XI; 2B Image of Landsat 7; 2C Image of ERS2; 2D Image of Envisat. The surrounded zones show rice fields that are not detectable with Spot, but could be detected by Radar images.

For Amborompotsy, the use of SAR and ASAR images was limited for definition of the training sites; the determination of class allowed a mask to be generated for areas identified as moors with which vegetation and rice fields might be confounded; the crest remained unchanged between the two dates. Figure 2 shows a part of Amborompotsy illustrating the difficulty of distinguishing rice fields from moor with optical data, and the possibility of masking moor with radar data (dark zone with low backscatter coefficient). The mask was obtained from comparisons of the backscatter coefficients between the two dates of data acquisition.

For Fenoarivo, the spectral response by digital number and calculated backscatter coefficients increased significantly between January and July for vegetation (p < 10^-6^) whereas they decreased for rice fields (p < 10^-9^); river systems had a low spectral response for both periods. Overall map accuracy for the six study areas was between 97 and 99.9%. Rice field map accuracy was between 82.3% and 100%. The Kappa coefficients were from 0.86 to 0.99 (table 1).

**Table 1:** Supervised classification results

Zones	Global map accuracy (%)	Rice fields map accuracy (%)	Kappa coefficient
z1	97	97.4	0.85
z2	98.7	99.0	0.94
z3	99.5	100	0.99
z4	99.9	82.3	0.99
z5	97.2	83.0	0.86
z6	97.9	90.5	0.96

### Entomological results

*An. funestus *is the main vector of malaria on the highlands of Madagascar; they make up 49% of mosquitoes in human landing collections and 97.2% in residual spray collections. The entomological study showed that mosquito aggressiveness varied from 0 to 20.5 bites/person/night with a mean of 3.87 bites/person/night. Vector abundance varied from 0 to 26.9 mosquitoes/house/night (table 2). The mosquito infectivity rate found by the CSP test was 0.3% of human landing collections and 0.04% of residual resting mosquito fauna.

**Table 2:** entomological results for *Anopheles funestus *collections

**Zone**	**Villages**	**Mosquito aggressiveness (number of bites/human/night)**	**Vector abundance (number of mosquitoes/house/night)**
Zone 1	Kiangara	8.92	15.20
	Mahatsinjo	6.50	6.55
	Ampotaka	16.42	26.95
	Manjakavaradrano	3.20	26.15

Zone 4	Anosilava	0	0
	Vatotokana	0.01	0.06
	Angodongodona	0.80	0.22
	Ankadidisa	0	0
	Isaha	0.18	0.24
	Ilomay	0.95	1.29
	Iarintsena	0	0

Zone 5	Mahasoa	3.21	0.25
	Ambatohirika	0.67	0.10
	Manara	0.12	0.06
	Mandrapakaholo	0.33	0.25
	Soaniarea	0.04	0.20
	Sahanala	2.46	0.36
	Androtsy	0.04	0

Zone 6	Ankaditsiary	9.42	3.50
	Ambalamontana	17.75	43.92
	Manavotra	13.05	8.14
	Amborompotsy	20.50	2.93

The five of the 27 villages in which no mosquitoes were collected are not listed in the table. This concerns two villages of zone 2 and three villages of zone 3.

### Malaria risk map by MCE

The abundance of vectors increased significantly with temperature: the correlation coefficient was 0.599 (p < 0.001). A significant negative correlation was evidenced between vector abundance and human population density: the correlation coefficient was -0.551 (p < 0.003). These results allowed objective comparison of the factors and assignment of ratings (table 3): the factor weights were 0.5040 for temperature, 0.2482 for the population density, 0.1079 for both distance from rice fields and rice field index and 0.0321 for the time from the last indoor insecticide spraying. The consistency ratio was 0.04 (<0.10); this was acceptable and therefore not re-evaluated.

**Table 3:** Pair-wise comparison of factors

	Temperature average	Population density	Distances from rice fields	Surface of rice fields	Indoor insecticide pulverisation
Temperature average	1	3	5	5	9
Population density	1/3	1	3	3	7
Distances from rice fields	1/5	1/3	1	1	5
Surface of rice fields	1/5	1/3	1	1	5
Indoor insecticide pulverisation	1/9	1/7	1/5	1/5	1

Factors weight	0.5040	0.2482	0.1079	0.1079	0.0321

The altitude constraint was very important. Considering the altitude as a constraint scored the area of Ambohibary (z2) as free of risk. The whole zone is higher than 1,600 m. Both Mahatsinjo (z1) and Amborompotsy (z6) are mostly lower than 1,000 m altitude with abundant vectors. Zones at risk of malaria transmission, requiring indoor insecticide spraying were positioned on a risk gradient from 0 to 255. For visual convenience, the colour scale has been organised into eight classes from low to high risk.

**Figure 3:**
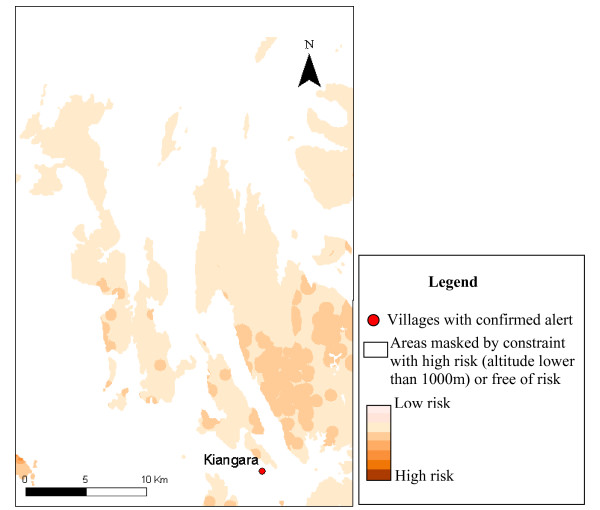
Malaria risk map of the z1(Mahatsinjo) with alert confirmation.

**Figure 4:**
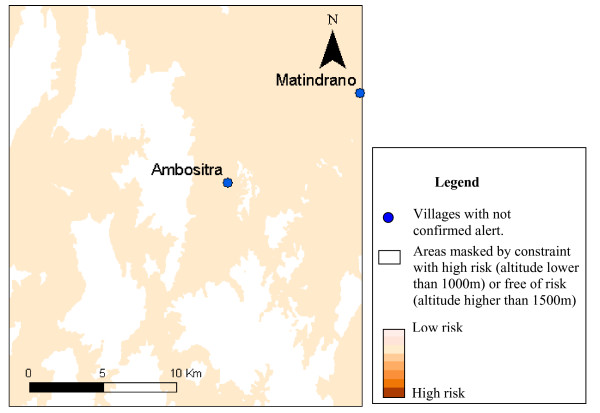
Malaria risk map of the z3 (Ambositra) with alert confirmation.

**Figure 5:**
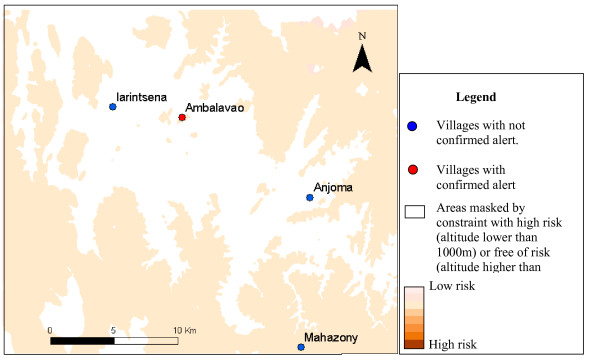
Malaria risk map of the z4 (Ambalavao) with alert confirmation.

**Figure 6:**
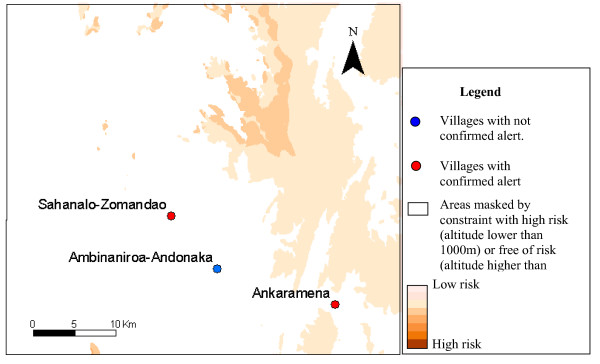
Malaria risk map of the z5 (Fenoarivo) with alert confirmation.

**Figure 7:**
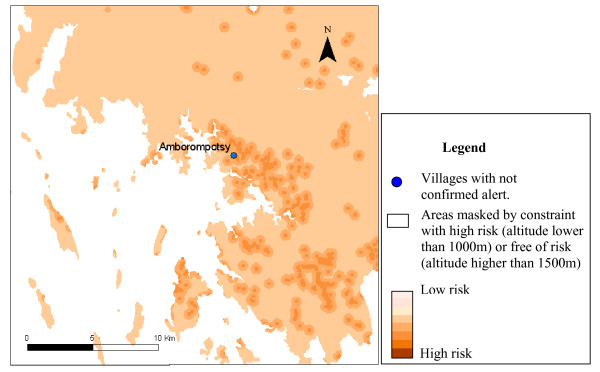
Malaria risk map of the z6 (Amborompotsy) with alert confirmation.

Only 13 of villages declaring alerts, covered by epidemiological surveillance posts, were included in the six study areas. The results of alert confirmation agreed with the risk map in nine cases (table 4). The figures 3 to 7 show the risk map and the validation with alerts confirmed by the epidemiological surveillance posts. The villages of Ambohibary and Antrafonomby are in zone 2 and were not mapped due to the altitude constraint.

**Table 4:** Risk map agreement

Villages	Constraint	Altitude = A (m)	Transmission	Epidemic risk	Status of alert confirmation	Risk map agreement
Kiangara	Yes	< 1000	Continuous	-	Confirmed	Yes
Ambositra	No	1000<A<1500	-	Low	Not confirmed	Yes
Matindrano	No	1000<A<1500	-	Low	Not confirmed	Yes
Ambalavao	Yes	A<1000	Continuous	-	Confirmed	Yes
Iarintsena	Yes	A<1000 m	Continuous	-	Not confirmed	No
Anjoma	Yes	A<1000	Continuous	-	Not confirmed	No
Mahazony	No	1000<A<1500	-	Low	Not confirmed	Yes
Sahanalo	Yes	<1000	Continuous	-	Confirmed	Yes
Ambinaniroa	Yes	<1000	Continuous	-	Not confirmed	No
Ankaramena	Yes	<1000	Continuous	-	Confirmed	Yes
Amborompotsy	No	1000<A<1500	-	High	Not confirmed	No
Ambohibary	Yes	A>1500	Absent	-	Not Confirmed	Yes
Antrafonomby	Yes	A>1500	Absent	-	Not confirmed	Yes

## Discussion

Predictions of vector presence and even abundance over large geographic areas are possible through the use of remotely sensed data [15-17]. Remote sensing is a valuable tool to visualize remote areas, and to localize rice fields from the satellite imagery. The advantage of radar imagery is that it can penetrate clouds, providing a solution for cloud-covered zones in tropical areas [18]. However, the exploitation of radar imaging can be limited by the mode of acquisition; topography can affect data acquisition in very uneven terrain. It has not been proven that the use of the backscatter coefficient is better than using the pixel value. Indeed, working with the absolute pixel value allowed us to fix the threshold for the extraction of moors.

Using only one type of image did not allow accurate detection of rice fields. The combination of the information obtained with the classification of Spot images, Radar image thresholds, visual interpretation and fieldwork improved the map quality [19]. A variety of factors prevented any one type of image from being satisfactory: the topography of landscape, the polymorphism of the radiometric response of rice fields in different states of development during the data acquisition, and confusion due to the reeds that colonize rivers in the absence of good irrigation techniques. However the available images obtained by various data acquisition techniques were used to correct the classification according to the diverse landscapes and situations.

Malaria is a vector-borne disease with environmental determinants: environmental changes influence malaria epidemic outbreaks [20]. A spatial approach has demonstrated the complexity of malaria transmission, and this complexity is such that we could not take all the factors linked to malaria transmission into account [21,22]. Onori and Grab listed direct and indirect factors of malaria transmission but most of them are not easily controllable [23]. Diuk-Wasser et al reported a paradoxical relationship between vector abundance and malaria transmission in irrigated rice field zones in Mali [24]. This finding incited us to focus on vector abundance and factors directly influencing malaria transmission. Altitude and temperature influence malaria transmission but we treated altitude as a constraint and so it was not weighted [25,26]. As altitude is closely linked to temperature, it was only used to mask areas in which indoor insecticide spraying was not warranted. For areas at an altitude lower than 1,000 m, the malaria control programme involved other adapted measures, particularly distribution of insecticide-impregnated bed nets.

Increased human population density has a negative impact on larval ecology of anopheline mosquitoes [27]. This is primarily due to overcrowding, to elimination of breeding sites due to construction, or to pollution of the breeding places resulting in lower vector densities in urban centres.

An earlier study conducted in the southern part of the Highlands showed that rice field surface areas are significantly linked to the abundance of vectors as evaluated entomologically [11]. In California, Wood et al found that high-mosquito-producing fields were located in areas with a diversity of land use, including livestock pastures, whereas the low-producing fields were in areas devoted almost exclusively to the cultivation of rice [28]. We could not confirm this in our study areas. For security reasons, rice fields are close to most of the villages. The average distance between rice fields and the villages involved in the entomological study is less than 100 m. According to Carter et al, the disease is concentrated around particular mosquito breeding sites and transmission is limited to the areas close to them: in Africa this distance typically ranges from a few hundred metres to a kilometre and rarely exceeds 2 or 3 km [29].

Indoor insecticide spraying was considered to be protective for the following two years. Indeed, in the villages of Analaroa, Androvakely, Antanetibe and Ankazobe, a rapid increase of notified malaria cases was observed two years after the cessation of the last indoor insecticide spraying campaign [14]. There have been similar findings in Belize and Mexico, but with a lag of four years: malaria cases increased from 10% to 96% from 1988 to 1992. This increase may well be linked to the reduction in numbers of houses sprayed with insecticide [15].

The low proportion of infected mosquitoes observed was comparable to values in unstable zones where malaria epidemics can occur. In such situations, prevention is the main objective. Prevention involves actions to maintain the vector population at a very low level. Vector abundance may be considered as a valuable indicator of malaria risk in our situation.

Entomological results are debatable in the absence of repetition for confirmation, but in our study mosquito sampling was always repeated and was in all cases during the rainy season. This allowed the objective analysis of every transmission factor. The consistency ratio (<0.10) demonstrates the coherence of factor weights we generated by our original approach.

The small number of alert confirmations included in the study zones is insufficient for the predictive values of the method to be calculated.

For several years, malaria risk mapping has been viewed as an important tool for targeting malaria control programmes [15,30-32]. The environmental factors that control the distribution of the vector and the disease need to be clearly understood if accurate risk maps are to be drawn [33].

## Conclusion

In a country with limited resources, like Madagascar, where most health care and programmes depend on lenders, a geographic information system is a potentially valuable tool for decision-making and optimising interventions. Our study was aimed at improving the management of malaria vector control programmes – programmes that aim to reduce the vector population, and thereby prevent epidemics. Considering the history of indoor insecticide spraying campaigns conducted on the highlands, the spraying was done in rural communes located at an altitude between 1,000 and 1,500 m. In these cases the entire commune is sprayed. The maps we constructed, with the resolution of the images used, allow targeting within 'communes' according to a risk gradient. Thus, indoor insecticide spraying could be limited to these priority zones.

The development of the method for determining zones at risk facilitates rapid updating of the maps. Continuous monitoring of the variables to detect changes, and regular notification of malaria cases to validate risk maps, are both necessary to improve the assessment of the risk factors.

The accuracy of the tool in space and time depends on the availability of data. This led us to use data from satellite imaging to identify potential breeding sites for the vectors responsible for epidemics. The limits on the use of this tool at a larger scale depend on the sufficiency or otherwise of the data allowing extraction of the information necessary for integrating all the factors influencing malaria transmission.

There is substantial geographic and ecological diversity between different regions of the central highlands. In addition to this, the mechanism of transmission is very complex, so it would be difficult, and over-ambitious, to try to develop and apply a single model that is valid for all the 100,000 km^2 ^of the central highlands. Ecozoning of the highlands would be required to identify zones with the same ecological and environmental characteristics, and then apply the risk map to this scale [34].

## Competing interests

The author(s) declare that they have no competing interests.

## Authors' contributions

IJ, JBD and FA conceived and helped to design and coordinate the study. JBD, RJ and RRV performed the entomological study and analysis of the results. RF and RRV performed most of the study. RLP conceived the statistical study and interpretation of data. JPR performed the spatial interpretation of remotely sensed data. RF drafted the manuscript. All the authors read and approved the final manuscript.
